# Genetic diversity of *Streptococcus pneumoniae* causing meningitis and sepsis in Singapore during the first year of PCV7 implementation

**DOI:** 10.1038/emi.2014.37

**Published:** 2014-06-04

**Authors:** Elita Jauneikaite, Johanna Mary Carnon Jefferies, Nicholas William Vere Churton, Raymond Tzer Pin Lin, Martin Lloyd Hibberd, Stuart Charles Clarke

**Affiliations:** 1 Faculty of Medicine and Institute for Life Sciences, University of Southampton, Southampton SO16 6YD, UK; 2 Infectious Diseases, Genome Institute of Singapore, Singapore 138672, Singapore; 3 NIHR Southampton Respiratory Biomedical Research Unit, Southampton SO16 6YD, UK; 4 Centre for Biological Sciences, University of Southampton, Southampton SO17 1BJ, UK; 5 National Public Health Laboratory, Ministry of Health, Singapore 169854, Singapore; 6 London School of Hygiene and Tropical Medicine, London WC1E 7HT, UK; 7 Public Health England, Southampton SO16 6YD, UK

**Keywords:** capsular type, invasive pneumococcal disease, multilocus sequence typing, Singapore, *Streptococcus pneumoniae*, vaccine

## Abstract

*Streptococcus pneumoniae* is a major cause of sepsis, meningitis and respiratory disease worldwide. Pneumococcal conjugate vaccines (PCVs) have now been implemented in many countries worldwide, including Singapore. To evaluate the effectiveness of these vaccines, pneumococcal surveillance studies are required. Detailed and unified pneumococcal epidemiology data are currently scarce in South East Asia. Thus, we present data on invasive pneumococcal (IPD) isolates from Singapore that could assist in evaluating the effectiveness of pneumococcal vaccine in Singapore. One hundred and fifty-nine invasive pneumococcal disease isolates were received by the National Public Health Laboratory in Singapore between June 2009 and August 2010. Isolates were characterized using serotyping and multilocus sequence typing. Twenty-four different serotypes were found, the most common of which were 19A, 3, 7F, 23F, 6B, 14, 8 and 19F (in rank order). One hundred and two sequence types were observed, of which 38 were novel due to new alleles or new combinations of already existing alleles. Based on the Simpson's Index of Diversity, serotypes 3, 6B and 19A were the most genetically diverse. Novel sequence types were more prevalent among conjugate vaccine serotypes 3, 19F and 23F and non-conjugate vaccine serotype 8, serogroup 15 and in non-typable isolates. We have demonstrated considerable genetic diversity among invasive pneumococci before and during the widespread use of conjugate vaccines in Singapore. Approximately half of all novel IPD clones identified in this study were non-conjugate vaccine serotypes. Although PCVs would target the most common serotypes, the high genetic diversity in non-vaccine serotypes would require further surveillance studies.

## INTRODUCTION

*Streptococcus pneumoniae* is an important cause of sepsis, meningitis, pneumonia and otitis media. Invasive pneumococcal disease (IPD) is responsible for high rates of morbidity and mortality, especially in young children, the elderly and the immunocompromised. According to a report by the World Health Organization, between 700 000 and one million children aged less than five years die annually due to pneumococcal infection.^1^ Pneumococcal pneumonia is considered to be one of the major causes of childhood mortality in the developing world and of adult mortality worldwide.^2,3^

Studies investigating the prevalence of serotypes, genotypes and antibiotic-resistant pneumococcal isolates in IPD in Singaporean children and adult populations were reported previously.^4,5,6,7,8,9,10^ The seven-valent pneumococcal conjugate vaccine (PCV7)—Prevnar (Pfizer, New York, USA) covers serotypes 4, 6B, 9V, 14, 18C, 19F and 23F, and has been available on demand in Singapore since its approval in 2005^11^ and was added to the childhood immunization programme in October 2009.^11,12,13^ Other available pneumococcal vaccines are PCV10—Synflorix (GlaxoSmithKline, Bentford, UK) that includes PCV7 serotypes and serotypes 1, 5 and 7F; and pneumococcal polysaccharide vaccine (PPV-23)—Pneumovax 23 (Merck, New Jersey, USA) which consists of pure polysaccharides of 23 pneumococcal serotypes (1, 2, 3, 4, 5, 6B, 7F, 8, 9N, 9V, 10A, 11A, 12F, 14, 15B, 17F, 18C, 19A, 19F, 20, 22F, 23F, 33F). PPV-23 is recommended by Ministry of Health in Singapore for elderly and people at higher risk of acquiring pneumococcal disease.^14^ In December 2011, PCV13—Prevnar 13 (Pfizer) which includes all PCV7 serotypes and the additional six serotypes of pneumococci: 1, 5, 7F, 3, 6A and 19A—replaced PCV7 in children immunization program in Singapore. The reported vaccine uptake was low – 21.6% in 2009 and 41% in 2010.^15^ This calls for further surveillance studies to document the true changes in prevalence of pneumococcal serotypes and possible vaccine serotype replacement by non-vaccine serotypes as it has been reported in other countries.^16^ Knowledge of genotypic and serotype data can also provide information on serotype replacement and vaccine escape isolates. For example, after PCV7 was implemented in the United States, an increase in infections caused by serotype 19A was reported.^17,18^

Undertaking disease surveillance provides baseline data for monitoring the impact of a new vaccine.^19^ This is particularly important for pneumococcal disease as PCVs include only a limited number of known serotypes. Pneumococcal populations undergo temporal changes in clonal distribution in the absence of pressure from a vaccine.^8,19^ Certain genotypes and serotypes of the pneumococcus may have a higher invasive potential than others;^17,20,21,22^ therefore, an understanding of underlying population structure is informative for formulating vaccine policy. Characterizing pneumococci by multilocus sequence typing (MLST) assesses the genetic relatedness of isolates by comparing the allelic sequences of seven housekeeping genes.^23^

Here, we present a descriptive epidemiology of the serotype and sequence type composition of invasive pneumococcal isolates during the early stages of the PCV7 implementation in Singapore.

## MATERIALS AND METHODS

### Bacterial isolates

One hundred and fifty-nine *S. pneumoniae* isolates from cases of IPD were collected as part of the pneumococcal surveillance program by the National Public Health Laboratory in Singapore from June 2009 to August 2010. One hundred and fifty-seven bacterial isolates (99.3%) included information on age, gender, date the isolate was received at the National Public Health Laboratory, serotype and specimen. One isolate (0.7%) had no information on age and gender. No information on vaccination status was available except for one isolate (0.7%).

### Pneumococcal capsular typing

Capsular typing was carried out by trained laboratory staff using the Pneumotest kit (Statens Serum Institute, Copenhagen, Denmark) at National Public Health Laboratory, Singapore. A simplified chessboard system^24^ was adopted to determine 21 vaccine-related serotypes or serogroups. The test was carried out by mixing 4 µL of bacterial suspension (pure bacterial culture and sterile phosphate buffered saline) with 4 µL of antiserum on a microscope slide. A coverslip was placed over the suspension and the slide was examined under phase contrast microscope. A reaction was considered to be positive when swelling of the capsule or agglutination was seen. Serogroups 6, 7, 9, 18, 19 and 23 were further tested with specific factor antisera to determine their serotypes. Non-vaccine-related serotypes or serogroups (not included in the simplified chessboard system) were reported as non-typable.

All isolates were serotyped in parallel using the polymerase chain reaction (PCR) method described by the Centre for Disease Control and Prevention.^25^

### DNA extraction

*S. pneumoniae* DNA for PCR and MLST sequencing was extracted from an overnight culture (grown on Columbia blood agar (Oxoid, Basingstoke, UK), incubated at 37°C at 5% CO_2_ incubator) using the QIAamp DNA mini kit (Qiagen, Hilden, Germany) according to the manufacturer's instructions.

### Multilocus sequence typing

DNA extractions were sent to Qiagen Sequencing Services for MLST. Sequence types (STs) were assigned using the MLST website.^26^ New alleles and new STs were assigned by the MLST website curator.

### Data analysis

Data were analysed based on age of the patient, serotypes and MLST. MLST data were analysed using goeBURST (PHYLOViZ)^27^ with parameters set for: seven loci per isolate, six identical loci per group and minimum three single loci variants per subgroup. Simpson's Index of Diversity (SID) was calculated as previously described.^28^

## RESULTS

Twelve pneumococcal isolates were from children under the age of five years; two isolates were from children between 5 and 18 years old; 82 isolates were from adults between 18 and 64 years old and 60 were from older adults (65 years old and above); one isolate had no age information. Ninety-four percent of all isolates (*n*=146) came from blood samples; the remainder were from cerebrospinal fluid (*n*=4), pleural (*n*=3) and peritoneal (*n*=2) fluids.

MLST indicated that two of 159 isolates were not *S. pneumoniae* and were excluded from further analysis (these two isolates were reported as non-typable by Pneumotest). Another two isolates did not yield a full MLST profile; therefore, they were excluded from MLST analysis.

### Serotype distribution

**Figure 1:**
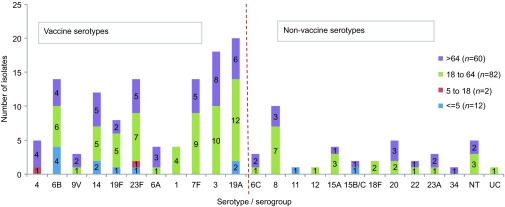
Serotype incidence among age groups. Total number of isolates 156. Vaccine coverage would be: PCV—38% (*n*=60, including the cross-protection for serotype 6A), PCV10—50% (*n*=79) and PCV13—74% (*n*=116). NT, non-typable by Pneumotest kit or mPCR method; UC, uncapsulated (*cpsA* not present, *ply* gene present, MLST indicated *S. pneumoniae*).

Isolates were assigned to 24 different serotypes and serogroups. The most common serotypes were serotype 19A (*n*=20), 3 (*n*=18), 7F (*n*=15; including one isolate with unknown age), 23F (*n*=14), 6B (*n*=14), 14 (*n*=12), 8 (*n*=10) and 19F (*n*=8) (Figure 1). Serotypes 6B, 19A and 14 were the most common in children under the age of five years old (Figure 1). The most common serotypes in the adult group (aged from 18 to 64 years inclusive) were 19A, 3, 7F, 8 and 23F (Figure 1). Serotypes 3, 19A, 23F, 14 and 7F were most commonly found in people that were aged 65 years and over (Figure 1).

This serotype data suggest the coverage by PCVs to be: PCV7—38% (*n*=60, including cross-protection for serotype 6A^29^), PCV10—50% (*n*=79), PCV13—74% (*n*=116) and coverage by PPV-23 would be 86% (*n*=134).

### Clonal diversity

A total of 102 STs were found, of which 38 STs were previously not reported to the MLST database (Table 1). Twenty-one of the new STs resulted from a new combination of existing alleles and 17 were due to new alleles (two isolates shared one new allele and had a different new allele). Eighteen new sequences for alleles were spread across: *aroE* (*n*=1), *gdh* (*n*=2), *gki* (*n*=2), *recP* (*n*=1), *spi* (*n*=2), *xpt* (*n*=7) and *ddl* (*n*=3).

**Figure 2:**
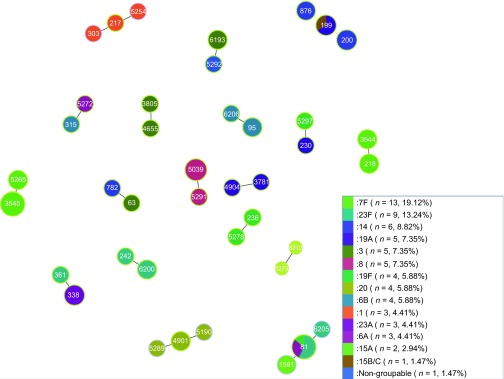
goeBURST (PHYLOViZ) analysis of 18 CCs. Population snapshot based on goeBURST analysis of 155 pneumococcal isolates. 18 CCs were formed. Each colour represents a different serotype.

**Figure 3:**
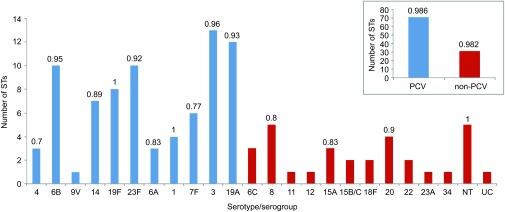
Clonal diversity. Total number of isolates *n*=155; 102 STs; 24 serotypes/serogroups. Clonal diversity within serotype/serogoup. Number above the column is SID. No SID value shows that three or less isolates were available of that serotype/serogroup. A slightly greater clonal diversity was within PCV-vaccine serotypes (number of different STs *n*=71) than within non-PCV—non-vaccine serotypes (number of different STs *n*=31). NT, non-typable by Pneumotest kit and mPCR method; UC, uncapsulated (*cpsA* not present, *ply* gene present, MLST indicates *S. pneumoniae*).

Our results showed that only few STs were shared between conjugate vaccine (PCV7 or PCV13) and non-conjugate vaccine serotypes (Table 1 and Figure 2) and that newly identified STs were more prevalent in conjugate vaccine serotypes. The clonal diversity was determined by SID for each serotype (Figure 3). Serotype 19F (*n*=8, STs=8) and non-typable isolates (*n*=5, STs=5) were the most diverse (SID value of 1), followed by serotypes 3 (*n*=18, STs=13) and 6B (*n*=14, STs=10) had the SID value 0.96 and 0.95, respectively (Figure 3). The novel STs were more prevalent among conjugate vaccine serotypes 3, 19F and 23F and non-conjugate vaccine serotype 8, serogroup 15 and in non-typable isolates (Table 1). The SID value differed only by 0.004 between PCV (*n*=115, STs=71) and non-PCV (*n*=40, STs=31) isolates (Figure 3). ST9, ST81 and ST320 were found in all three age groups (children under 18, 18–64 and over 64 years). ST9 was consistently found only in serotype 14 whilst ST81 was found in 6A, 19F and 23F. One of the new STs, ST6197, was identified in both adult groups (Table 1).

### Pneumococcal Molecular Epidemiology Network (PMEN) clones

**Figure 4:**
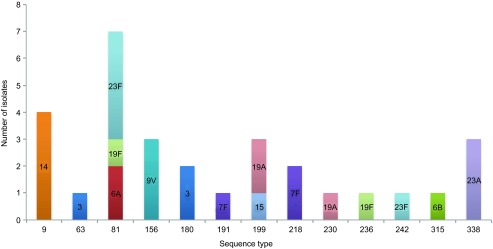
The same STs as PMEN clones within Singaporean isolates (*n*=31). Serotypes are shown next to the corresponding ST.

Thirty-one of the 155 isolates had the same MLST allelic profiles as 12 globally disseminated antibiotic resistant clones reported to the PMEN^30^ (Figure 4). The most common STs were ST81 and ST199; the same two STs were the only one to be found in more than one serotype in this dataset (Figure 4). The potential PMEN clones in our dataset had the same serotypes as multidrug resistant clones reported to PMEN website and these isolates were found in all age groups (Table 1). Unfortunately, phenotypic antibiotic susceptibility data were not available for this set of isolates. The whole genome sequences were available (Jauneikaite E *et al.*, unpublished data) and used to identify presence or absence of the specific antibiotic resistance genes for the seven antibiotics used by the PMEN to identify multidrug resistant strains: penicillin, erythromycin, clindamycin, tetracycline, co-trimoxazole, cefotaxime and chloramphenicol. For the clone to be classified as an international antibiotic resistant pneumococcal clone, the isolate needs to be resistant to one or more of antibiotics of wide clinical use.^30^ Sixteen isolates had all three *pbp2A*, *pbp1A* and *pbp1B* alleles present, the other ten isolates carried either *pbpX*, *pbp2X* and *pbp2B* or a combination of any two of these alleles (Jauneikaite E *et al.*, unpublished data). In total, eleven isolates also had macrolide resistance alleles (*mefA*, *emrB* and *msrD*) (Jauneikaite E *et al.*, unpublished data). Additionally, seven isolates had chloramphenicol resistance allele *cat(pC194)* and sixteen isolates had tetracycline resistance allele—*tet(M)* (Jauneikaite E *et al.*, unpublished data). Overall, every potential PMEN clone had resistance alleles to at least three different antibiotics.

### goeBURST analysis

goeBURST^27^ showed the results for all 155 isolates (Figure 2). Sixty-two out of 102 were singletons and 18 clonal complexes (CCs) were formed (Figure 2).

Comparative eBURST^26^ of our 155 isolates against the whole *S. pneumoniae* MLST database showed that all of the 102 STs fall into CCs and were distributed across the eBURST diagram of the whole *S. pneumoniae* MLST database. The highest number of STs (*n*=11, 18 isolates) fell into CC156. Four CCs consisted of STs found in this study, of which one CC was solely formed by ST6212 and ST5273. No singletons were found (last checked 31 May 2013).

Seven of the 18 CCs consisted of two or more different serotypes with different STs that differ just by one locus (Figure 2). ST81 and ST199 were from diverse serotypes (Figure 2).

## DISCUSSION

In this 2009–2010 study, undertaken in the first year of PCV7 implementation in Singapore, we found that 74% of the serotypes isolated are included in PCV13 and 38% are included in PCV7. The fact that only 38% of isolates identified were PCV7 serotypes (plus serotype 6A) suggests that these serotypes represent a smaller proportion of potential IPD-causing serotypes and that vaccination with the PCV13 may increase protection against IPD. The low percentage of PCV7-related serotypes could be due to the herd immunity effect, as reported in the United States^31,32^ and United Kingdom,^33^ however, considering that the uptake of PCV7 is low in Singapore.^15^ Also, minimal changes were reported in the incidences of IPD in children in Singapore after early stages of PCV7 implementation during 2005–2010 by Thoon *et al*.^34^

Within our dataset, the most common serotypes were 3, 6B, 7F, 8, 14, 19A, 19F and 23F. All but serotype 8—are included in PCV13. PCV13 was implemented in the childhood immunization schedule in Singapore in December 2011. The National Public Health Laboratory and KK Women & Children's Hospital in Singapore, reported to the Ministry of Health in Singapore that the most common pneumococcal serotypes found in IPD cases in year 2010–2011 in paediatric cases (exact age not reported) were serotypes 19A (40%), 14 (15%) and 6B (13%), while in adults, the diversity of serotypes found was greater and the most common found were: serotype 3 (13%), 14 (10%), 19A (10%) and 8 (10%).^10^ Later, Thoon *et al.*^34^ reported the changes in most common serotypes found in children population in KK Women & Children's hospital, highlighting the increase in invasive disease caused by serotypes 19A. In our dataset, only 12 IPD isolates from children under the age of five years old were available, most common being 6B, 19A and 14. These data are in agreement with previous reports from the region.^12,35^

Most of the 102 STs identified here have been reported previously on the MLST website; however, a substantial number (*n*=38, 37%) were new STs. Serotype 19A was the most common serotype found in our study. One pneumococcal isolate ST320^19A^ was isolated from pleural fluid sample from a patient under the age of five years, who was vaccinated with four doses of PCV7 which does not include serotype 19A. Unfortunately, no further clinical information was available for this patient. It has been reported that ST320 is commonly associated with serotype 19A and this clone is widely spread across the world.^26^ The penicillin resistant ST320 serotype 19A clone was commonly found to cause IPD in children in the USA between 2005 and 2007.^36^ Another common clone of serotype 19A is ST199. This ST199 is reported as the major genotype for penicillin susceptible invasive serotype 15B/C and intermediately penicillin resistant serotype 19A in the United States.^36^ In our study, one ST199 isolate was serotype 15B/C in the adult group over 64 years old. None of the other serotype 19A isolates belonged to any of the new ST or other major antibiotic resistance CCs. Here we identified four isolates as ST320^19A^ and two isolates as ST199^19A^. ST320^19A^ was reported to be the dominant clone in Asian countries,^37^ but an increase in different and new (previously not reported to MLST database) genotypes are also seen.^37^

We have demonstrated considerable genetic diversity among Singaporean IPD isolates using serotyping and MLST. Approximately half of all novel IPD clones identified in this study were non-conjugate vaccine serotypes. There is scarce information available on the molecular epidemiology of pneumococcal disease not only from Singapore, but also from other South East Asian countries. Increasing availability and affordability of high throughput sequencing technologies has the potential to provide more information on circulating strains in different countries and will help to map out possible transmission routes globally.

In order to evaluate the true effects of pneumococcal conjugate vaccines in Singapore, studies investigating the molecular epidemiology of disease-causing pneumococci are in progress. However, pneumococcal carriage is a precursor to pneumococcal diseases; therefore, we would strongly encourage pneumococcal carriage studies to be undertaken in order to assess the circulating pneumococcal population in Singapore.

## Figures and Tables

**Table 1:** ST and serotype/serogroup distribution within age groups

Serotype/serogroup ST	
**Children: ≤5 years** (*n*=12)	
Serotype 6B	315 (*n*=1); 2782 (*n*=1); **6198** (*n*=1); unavailable (*n*=1)
Group 11	**6191** (*n*=1)
Serotype 14	9 (*n*=1)
Serotype 15B/C	**6192** (*n*=1)
Serotype 19A	320 (*n*=2)
Serotype 19F	236 (*n*=1); 320 (*n*=1)
Serotype 23F	311 (*n*=1)
**Children: 6–18 years** (*n*=2)	
Serotype 4	**5275** (*n*=1)
Serotype 23F	81 (*n*=1)
**Adults: 19–64 years** (*n*=82)	
Serotype 1	217 (*n*=1); 303 (*n*=1); 5044 (*n*=1); **5254** (*n*=1)
Serotype 3	458 (*n*=3); 505 (*n*=1); 4389 (*n*=1); **6193** (*n*=2); 3805 (*n*=1); **63** (*n*=1)
Serotype 6A	5832 (*n*=1)
Serotype 6B	386 (*n*=1); 1518 (*n*=3); 2779 (*n*=1); **6206** (*n*=1)
Serotype 6C	**6209** (*n*=1)
Serotype 7F	218 (*n*=1); 3545 (*n*=5); **5265** (*n*=2); 5441 (*n*=1)
Serotype 8	5039 (*n*=4); **5291** (*n*=1); **6197** (*n*=2)
Serotype 9V	156 (*n*=1)
Serogroup 12	989 (*n*=1)
Serotype 14	9 (*n*=1); 143 (*n*=1); 200 (*n*=1); 876 (*n*=1); **6196** (*n*=1)
Serotype 15A	1591 (*n*=1); **6202** (*n*=1); **6204** (*n*=1)
Serogroup 18F	291 (*n*=1); 2711 (*n*=1)
Serotype 19A	230 (*n*=1); 320 (*n*=2); 416(*n*=3); 847 (*n*=1); 1848 (*n*=1); 2013 (*n*=2); 3781 (*n*=1); 3111 (*n*=1)
Serotype 19F	9 (*n*=1); **5260** (*n*=1); **5278** (*n*=1);3791 (*n*=1); **6199** (*n*=1); 5297 (*n*=1)
Serogroup 20	4901 (*n*=1); 5190 (*n*=1)
Serogroup 22	**6194** (*n*=1)
Serotype 23A	338 (*n*=1)
Serotype 23F	81 (*n*=3); 361 (*n*=1); **6195** (*n*=1); **6200** (*n*=2)
Non-typable	**6207** (*n*=1); **6210** (*n*=1); 5292 (*n*=1)
Uncapsulated	**6201** (*n*=1)
**Adults: 65 years and above** (*n*=60)	
Serotype 3	180 (*n*=2); 4655 (*n*=1); 4909 (*n*=1); **5255** (*n*=1); **5267** (*n*=1); **5299** (*n*=1)
Serotype 4	4127 (*n*=3); 5872 (*n*=1)
Serotype 6A	81 (*n*=2); **5272** (*n*=1)
Serotype 6B	95 (*n*=2); 3114 (*n*=1); 5241 (*n*=1)
Serotype 6C	224 (*n*=1); **5295** (*n*=1)
Serotype 7F	191 (*n*=1); 218 (*n*=1); 3544 (*n*=2); 3545(*n*=1)
Serotype 8	2234 (*n*=1); **6197** (*n*=1); **6203** (*n*=1)
Serotype 9V	156 (*n*=2)
Serotype 14	9 (*n*=2); 200 (*n*=2); 782 (*n*=1); 876 (*n*=1)
Serotype 15A	1591 (*n*=1)
Serotype 15B/C	199 (*n*=1)
Serotype 19A	199 (*n*=2); 2013 (*n*=1); 2102 (*n*=1); 4904 (*n*=1); 4937 (*n*=1)
Serotype 19F	81 (*n*=1); 320 (*n*=1)
Serogroup 20	235 (*n*=1); 4901 (*n*=1); **5289** (*n*=1)
Serogroup 22	433 (*n*=1)
Serotype 23A	338 (*n*=2)
Serotype 23F	242 (*n*=1) **5273** (*n*=1) **5296** (*n*=1) **6205** (*n*=1) **6212** (*n*=1)
Serotype 34	2832 (*n*=1)
Non-typable	5893 (*n*=1); **6208** (*n*=1)

Non-typable by Pneumotest and mPCR methods.

In bold: novel STs assigned in this study.

One pneumococcal sample did not generate MLST profile.
